# Astragalin-induced cell death is caspase-dependent and enhances the susceptibility of lung cancer cells to tumor necrosis factor by inhibiting the NF-κB pathway

**DOI:** 10.18632/oncotarget.15264

**Published:** 2017-02-10

**Authors:** Minghui Chen, Fangfang Cai, Daolong Zha, Xueshi Wang, Wenjing Zhang, Yan He, Qilai Huang, Hongqin Zhuang, Zi-Chun Hua

**Affiliations:** ^1^ The State Key Laboratory of Pharmaceutical Biotechnology, School of Life Sciences, Nanjing University, Nanjing, China; ^2^ State Key Laboratory of Quality Research in Chinese Medicines, Macau University of Science and Technology, Avenida Wai Long, Taipa, Macau; ^3^ Changzhou High-Tech Research Institute of Nanjing University and Target Pharma Laboratory, Changzhou, Jiangsu, China; ^4^ College of Pharmacy, Nanjing University of Chinese Medicine, Nanjing, China

**Keywords:** natural compounds, astragalin, apoptosis, non-small cell lung cancer, tumor therapy

## Abstract

Flavonoids are naturally occurring polyphenolic compounds and are among the most promising anticancer agents. Here, we demonstrate that the flavonoid astragalin (AG), also known as kaempferol-3-O-β-D-glucoside, induces cell death. This was prevented by the caspase inhibitors z-DEVD-FMK and z-LEHD-FMK. AG-induced cell death was associated with an increase in the Bax:Bcl-2 ratio and amplified by the inhibition of extracellular signal-regulated kinase (ERK)-1/2 and Akt signaling. Meanwhile, AG suppressed LPS-induced NF-κB activation. Additional studies revealed that AG inhibited tumor necrosis factor-alpha (TNFα)-induced NF-κB activity. AG also potentiated TNFα-induced apoptosis in A549 cells. Furthermore, using a mouse xenograft model, we demonstrated that AG suppressed tumor growth and induced cancer cell apoptosis *in vivo*. Taken together, these results suggest that AG may be a promising cancer therapeutic drug that warrants further investigation into its potential clinical applications.

## INTRODUCTION

Many antitumor compounds kill sensitive cells by inducing apoptosis. Generally, apoptosis pathways include signaling *via* mitochondria (intrinsic) or the death receptors (extrinsic) pathway. The extrinsic pathway is initiated by the ligation of death receptors (DRs), e.g., Fas or TNF receptors. Activated initiator caspase-8 then cleaves the downstream effector caspases (caspase-3, -6 and -7) in a caspase cascade, which subsequently cleave a broad spectrum of target substrates, resulting in apoptotic cell death. The intrinsic pathway includes alterations in mitochondrial permeability transition as well as mitochondrial membrane potential, which results in the discharge of apoptogenic factors, e.g., apoptosis-inducing factor (AIF) and cytochrome c, from the mitochondria into the cytosol [1]. In both the intrinsic and extrinsic pathways, caspase-3 is in charge of the cleavage of poly (ADP-ribose) polymerase (PARP) during apoptosis [2]. Apoptosis is an intricate process; it is mediated by a number of molecules that work to either inhibit (including Bcl-xl, Bcl-2, and the IAP family of proteins) or promote (such as Bak, Bax, and caspases) cell death [3]. A common phenomenon of many cancers is defective apoptosis, which is also a key factor in tumorigenesis as well as in treatment resistance; therefore, apoptotic pathways are often targets of cancer therapies.

Cancer is still the main cause of mortality globally. Despite advances in the development of new therapeutic options for cancer, chemotherapy is still the fundamental tool for cancer treatment; it functions primarily by inducing cancer cell apoptosis. The leading cancer death worldwide is lung cancer; non-small cell lung cancer (NSCLC) is responsible for more than 80% of total lung cancer cases [4, 5]. Approximately 30-40% of patients present with locally advanced stage III disease [6]. Many cancer patients remain refractory to therapy even though there have been significant improvements in chemotherapy, radiation therapy and surgery. Thus, it is necessary to identify new agents that can improve the antitumor effects and minimize the side effects of commonly prescribed chemotherapy drugs.

**Figure 1:**
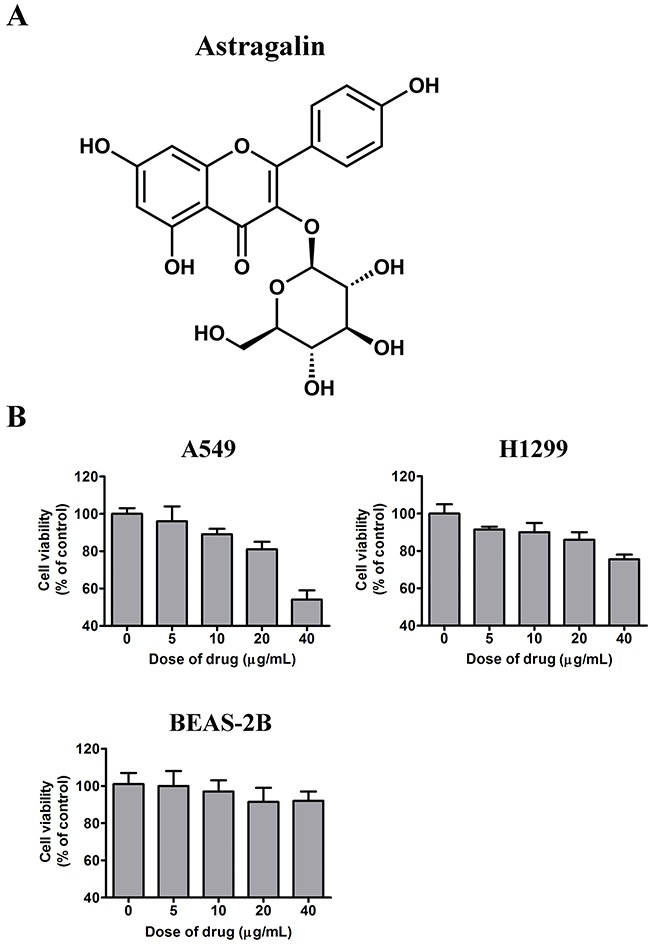
Effects of AG on the growth of NSCLC cells *in vitro* **A**. Chemical structure of AG. **B**. A549, H1299, and BEAS-2B cells were treated with AG at different concentrations (0, 5, 10, 20, 40 μg/mL) for 24 h, and the cell viability was assessed by MTT assay. *Each bar* shows the mean ± SD of three independent experiments, performed in triplicate.

Nowadays, traditional Chinese herbal remedies have been given significant scrutiny as new anticancer drugs and novel chemotherapy adjuvants to improve the competence of cancer chemotherapy and to relieve chemotherapy side effects. Despite the fact that the healing mechanisms are not yet understood, some agents have aided cancer patients battling their disease, resulting in fewer side effects than other treatments [7]. Chemicals taken from herbs have potential because several natural compounds, which include a selection of flavonoid compounds, have been shown to exhibit antitumor functions [8, 9]. Cancer research trends have shown that flavonoids can be used alone or in combination with other therapeutic agents to control the growth of many types of tumor cells [10]. Astragalin (AG; C_21_H_20_O_11_; shown in Figure 1A), also known as kaempferol-3-O-β-D-glucoside, is a flavonoid isolated from the leaves of persimmon or Rosa agrestis. It is widely found in tea and has been used to treat many diseases as a traditional Chinese medicine for a long time. Several groups have confirmed that AG exhibits a number of biological properties, including anti-inflammatory, antioxidant, and anti-atopic dermatitis effects [11–14]. In addition, AG can attenuate lipopolysaccharide (LPS)-induced inflammatory responses by suppressing the NF-кB signaling pathway [15]. However, few studies have investigated the therapeutic potential of AG as a cancer therapy agent. Herein, we investigated the effects of this compound on cell viability as well as apoptosis induction in human lung cancer cell lines. We also assessed whether the MAPK cascade, caspase activation and NF-кB pathway are involved in the underlying mechanisms. Additionally, our data demonstrated that AG could sensitize tumor cells to TNFα-triggered cell death *via* inhibiting the activity of NF-кB. Meanwhile, the results demonstrated that AG could, in a time-dependent manner, alter the sensitivity of NSCLC cell line A549 to Fas/FasL-induced apoptosis.

## RESULTS

### Effects of AG on NSCLC cell growth

Firstly, MTT assay was carried out to evaluate the effects of AG on tumor cell proliferation. A normal cell line and two NSCLC cell lines were treated with AG for 24 h. The growth of the NSCLC cell lines A549 and H1299 was significantly inhibited dose-dependently, as shown in Figure 1B. In A549 cells, growth was inhibited by 3.2 ± 0.74% and 41.2 ± 2.55% with AG treatment at 5 μg/mL and 40 μg/mL, respectively. In contrast, H1299 cells were relatively resistant to AG treatment; the rate of inhibition was only 13.2 ± 0.64% after treatment with 20 μg/mL of AG. However, we detected no obvious cytotoxicity in the BEAS-2B cells. These observations suggested that AG exhibited selective cytotoxic effects on tumor cells without exerting cytotoxic effects on normal cells.

### Effects of AG on NSCLC cell apoptosis

**Figure 2:**
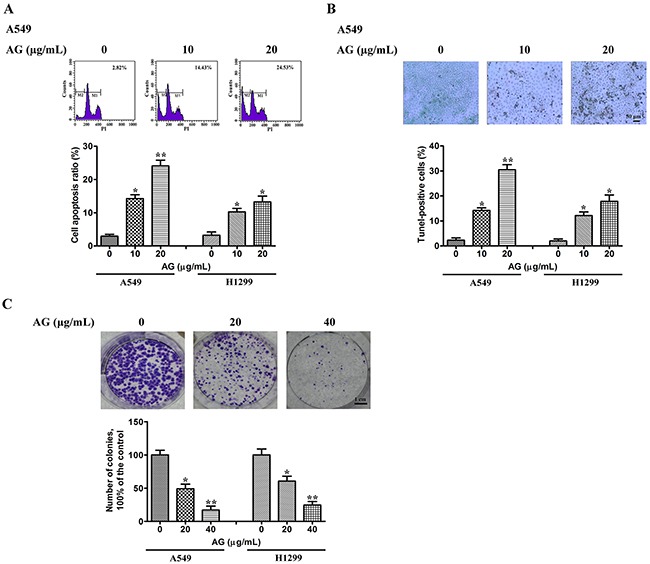
Effects of AG on NSCLC cell apoptosis **A**. A549 and H1299 cells were exposed to AG at different concentrations (0, 10, 20 μg/mL). 18 h later, all cells were harvested for flow cytometry analysis. PI-stained cells were analyzed with the percentages of apoptosis cells shown. The experiments were carried out independently in triplicate; representative data are shown. *p < 0.05, **p < 0.01. PI staining profile of A549 cells is also included. **B**. A549 and H1299 cells were exposed to AG at different concentrations (0, 10, 20 μg/mL) for 18 h. TUNEL assays were performed according to the manufacturer's instructions. The rate of apoptosis was expressed as the percentage of total cells counted. *Each bar* shows the mean ± SD of three independent experiments, performed in triplicate. *p < 0.05, **p < 0.01. TUNEL staining profile of A549 cells is also shown. A *dark brown* DAB signal indicates positive staining, while shades of *blue-green* to *greenish tan* signifies a non-reactive cell. **C**. Colony formation ability of NSCLC cells treated with AG at different concentrations (0, 20, 40 μg/mL). *Each bar* shows the mean ± SD of three independent experiments, performed in triplicate. *p < 0.05, **p < 0.01. Representative dishes of A549 cells evaluated by colony-forming assay are also included.

Next, experiments were performed to conclude whether there was a close association between AG-mediated anti-proliferative effects on cells and apoptotic cell death. Propidium iodide (PI) staining was used for the evaluation of apoptosis. The NSCLC cell lines A549 and H1299 were treated with AG at the indicated concentrations. As shown in Figure 2A, the number of cells stained with PI increased after AG treatment dose-dependently, indicating the induction of apoptosis. DAPI staining also indicated the induction of apoptosis in A549 cells treated with AG. Apoptotic cells show a typical morphological change termed nuclear condensation. This change is able to be detected by DAPI staining, including in the early stages of cell death [16]. Most A549 cells in the untreated group showed fluorescence of a uniform blue color, as shown in Supplementary Figure 1. The percentage of cells that displayed condensed nuclei (naturally apoptotic cells) was 6.2 ± 0.6 (%). However, nuclear condensation was apparent in cells treated with 10 μg/mL AG; the ratio of cells exhibiting apoptotic morphology was 18.5 ± 2.1 (%). The results of TUNEL assays were consistent with the data described above (Figure 2B). Compared with untreated cells, 20 μg/mL AG induced apoptosis in 30.3% and 19.3% of A549 and H1299 cells, respectively.

### Effects of AG on NSCLC cell clonogenic growth

We further investigated whether treatment with AG had an effect on NSCLC cell clonogenic growth. Colony-forming assays showed that AG suppressed the clonogenic growth of H1299 and A549 cells dose-dependently. Treatment with 40 μg/mL AG significantly inhibited the clonogenic growth of H1299 and A549 cells by 72.9 % and 81.3%, respectively (Figure 2C).

### AG induces apoptosis in a caspase-dependent manner

**Figure 3:**
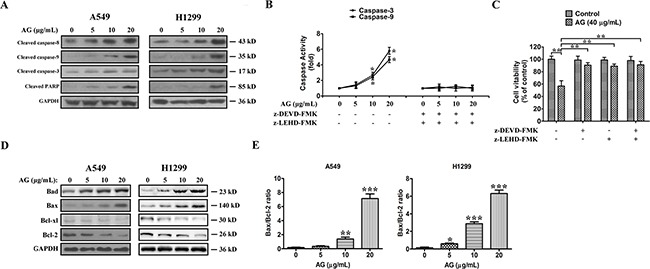
AG-induced apoptosis is mediated through the caspase-dependent apoptotic pathway in NSCLC cells **A**. Cleaved caspase-8, cleaved caspase-9, cleaved caspase-3, and cleaved PARP expressions in A549 and H1299 cells treated with AG at different concentrations (0, 5, 10, 20 μg/mL). **B**. Activity of caspase-3 and caspase-9 in A549 cells treated with AG at different concentrations (0, 5, 10, 20 μg/mL) for 24 h with or without caspase inhibitors. Data are presented as fold increases as determined by quantitative analysis. *p < 0.05. **C**. Viability of A549 cells after treatment with caspase inhibitors. Cells were treated with inhibitors for 2 h before the 24 h treatments, after which cell viability was determined by MTT assay. Data are representative of three independent experiments. **p < 0.01. **D**. Expressions of the Bcl-2 family proteins, Bcl-2, Bcl-xl, Bax, Bad, in A549 and H1299 cells treated with AG at different concentrations (0, 5, 10, 20 μg/mL) for 24 h. **E**. Band intensity was quantified by Image J software. The ratio of Bax:Bcl-2 was shown. The results shown are representative of three different experiments. Data are represented as mean ± SD, **p < 0.01, ***p < 0.001.

To investigate the ways by which AG induces apoptosis in NSCLC cells, cells were exposed to 5, 10, or 20 μg/mL AG for 24 h, and then caspase activation was analyzed using Western blotting. Apoptosis is mediated mainly by the caspase enzymes. Effector caspases, such as caspase-3, -6, and -7, are eventually activated when any stimuli triggers apoptosis. As a result of AG treatment, increased cleavage of caspase-8, -9, -3, and PARP were observed (Figure 3A). Figure 3B shows the measurements of caspase activity, which implied that caspase-9 and caspase-3 activities were increased 4.6- and 5.8-fold in cells receiving treatment of 20 μg/mL AG, as compared with the control. With the combination of caspase inhibitors z-LEHD-FMK and z-DEVD-FMK, the AG-induced activation of caspase was abolished, and A549 cells were prevented from apoptosis. Caspase inhibitors also increased cell viability after combined treatment (Figure 3C). Our results suggested that activation of caspase-mediated apoptotic pathway was a major mechanism by which AG exerted its apoptotic effects of NSCLC cells.

Then, the effects of AG treatment on the balance between pro-apoptotic (for example, Bad or Bax) and anti-apoptotic (for example, Bcl-xl or Bcl-2) members of the Bcl-2 family in H1299 and A549 cells were examined. The data indicated that the intrinsic apoptotic pathway was also activated, as demonstrated by a decrease in the expression of Bcl-xl and Bcl-2 and an increase in the expression of Bad and Bax (Figure 3D). Thus, the Bax:Bcl-2 ratio was increased in both NSCLC cell lines (Figure 3E). These data suggested that AG induced NSCLC cell apoptosis *via* caspase-dependent mitochondrial pathway.

### Inhibitory effects of AG on PI3K/Akt and MAPK activation

**Figure 4:**
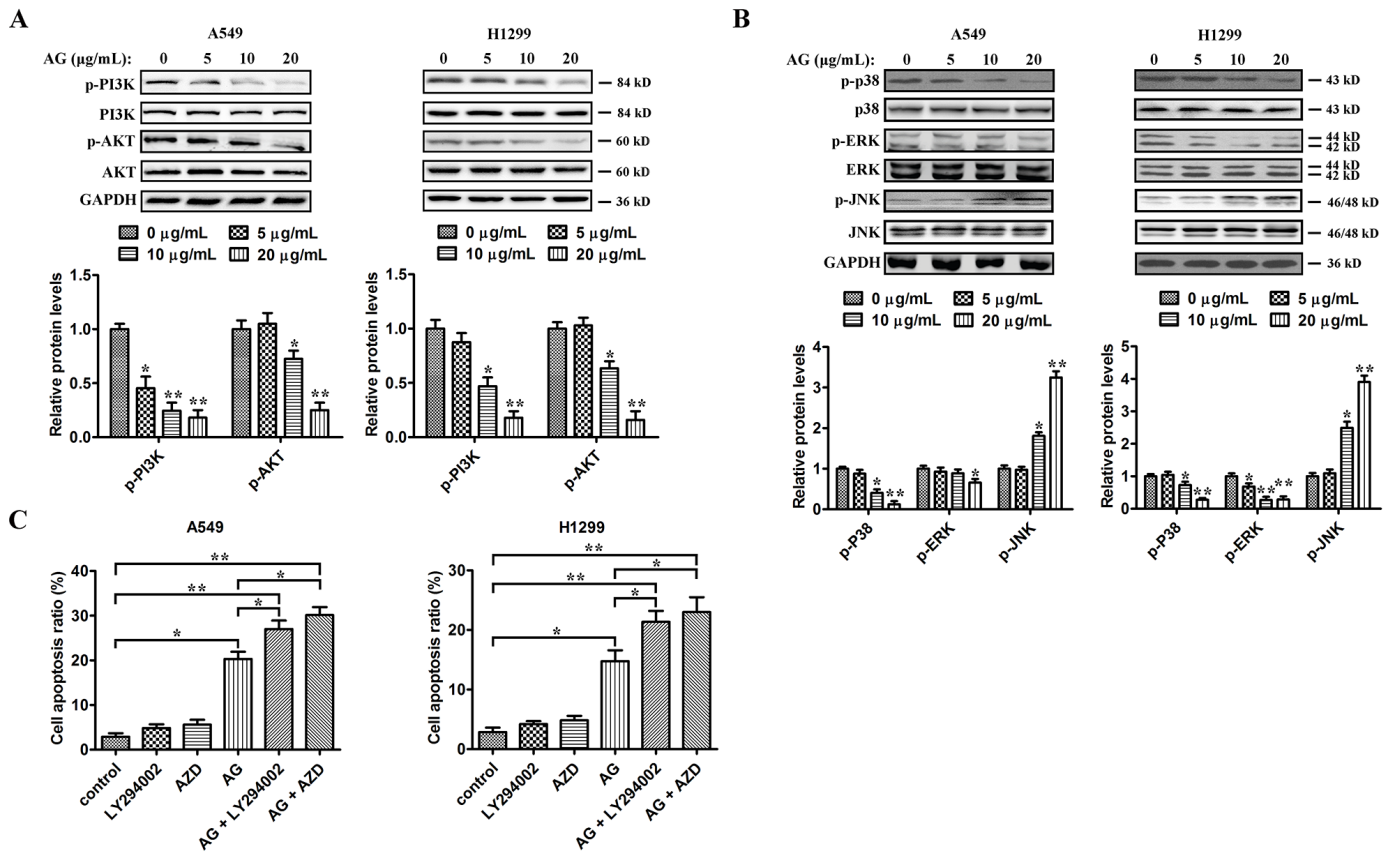
Effects of AG on PI3K/Akt and MAPKs signaling pathway A549 and H1299 cells were treated with AG at different concentrations (0, 5, 10, 20 μg/mL) for 24 h. **A**. Akt, p-Akt, PI3K, and p-PI3K proteins in whole cell lysates were determined with specific antibodies. GAPDH was used as loading control. **B**. Western blotting was performed to detect the levels of p-p38, p38, ERK, p-ERK, JNK, and p-JNK respectively. Densitometric quantification of the immunoblot data in (A) and (B) is also shown and data are represented as mean ± SD. *p < 0.05, **p < 0.01. **C**. A549 and H1299 cells were treated with AG (20 μg/mL), AZD6244 (AZD; 2 μM), LY294002 (5 μM), the combination of AG with AZD, or the combination of AG with LY294002 for 24 h before determination of cell death by flow cytometry analysis. Data are representative of three independent experiments. *p < 0.05, **p < 0.01.

We then assessed alterations in the survival pathways in NSCLC cells to further investigate the mechanisms behind AG-induced cell death. Mitogenic and Akt survival pathways have the ability to intensify cellular proliferation, inhibit apoptosis, and potentiate the downstream NF-κB survival pathway [17]. To assess whether the Akt pathway was affected by AG and played a role in AG-induced NSCLC cell death, A549 and H1299 cells were exposed to increasing dosages of AG for 24 h. As shown in Figure 4A, AG suppressed the activation of PI3K and Akt dose-dependently. Since c-Jun N-terminal kinase (JNK), ERK and p38^MAPK^ also pay key roles in determining cell fate, we also evaluated the effects of AG on the activation of these protein kinases. As shown in Figure 4B, AG decreased p38 and ERK phosphorylation dose-dependently in both cell lines, but increased JNK phosphorylation. Specific inhibitors were then used to assess whether the phosphorylation of Akt or ERK plays a critical role in AG-triggered cell death. Treating cells with AZD6244, a MEK inhibitor, or LY294002, a PI3K/Akt inhibitor increased AG-induced apoptosis in A549 and H1299 cells (Figure 4C). Thus, inhibition of the survival MAPK ERK in A549 and H1299 cells may also account for the apoptotic effects of AG.

### AG inhibits NF-κB p65 nuclear translocation and IκBα degradation

**Figure 5:**
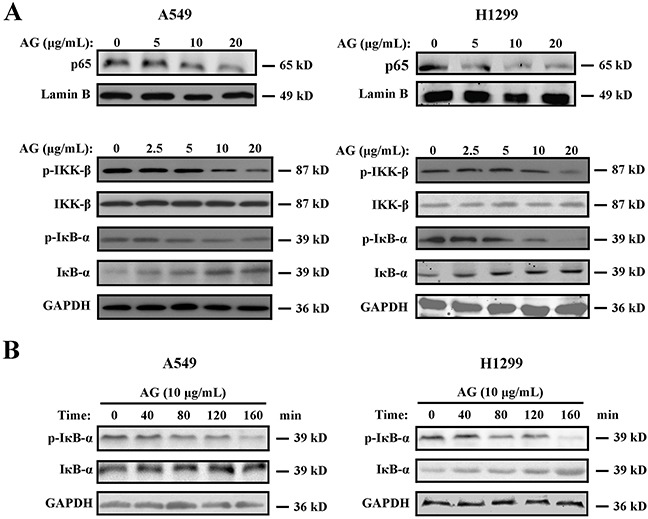
Effects of AG on the NF-κB pathway **A**. A549 and H1299 cells were treated with AG at different concentrations (0, 2.5, 5, 10, 20 μg/mL) for 24 h. Nuclear proteins were extracted and subjected to Western blotting for p65 detection. Lamin B was used as loading control. Additionally, the whole cell extracts with the same treatment were prepared and analyzed for p-IKK-β, IKK-β, IκBα and p-IκBα expression. GAPDH was used as loading control. **B**. A549 and H1299 cells were treated with AG at the concentration of 10 μg/mL for the indicated time. The whole cell extracts were prepared and analyzed for IκBα and p-IκBα expression. The results shown are representative of three different experiments.

NF-κB is a cellular survival factor, which can lead to the upregulation of several anti-apoptotic genes that block apoptosis, including c-FLIP, Bcl-xl, Bcl-2, and Mcl-1 [18]. Inhibition of NF-κB results in decreased expression of the NF-κB target anti-apoptotic proteins, thus promoting apoptosis. Thereafter, we investigated whether different concentrations of AG could affect the activity of NF-κB. Low doses of AG did not alter the protein level of NF-κB/p65 apparently. However, treatment of A549 and H1299 cells with ≥10 μg/mL AG inhibited the nucleus translocation of NF-κB (Figure 5A), leading to the inhibition of the transactivation of NF-κB-regulated genes, including *Bcl-xl* and *Bcl-2* (Supplementary Figure 2). Additionally, AG treatment resulted in increased IκBα levels in both a time- and dose-dependent manner (Figure 5A, 5B). AG treatment also suppressed the phosphorylation of IκBα, as shown in Figures 5A and 5B. This suggested that it abrogated the release of IκBα from NF-κB heterodimer and blocked the proteasomal degradation of IκBα. IκB phosphorylation by its kinase IκB kinase (IKK) is a crucial step in the activation of NF-κB [19]. We subsequently studied whether AG inhibited IKK activation in NSCLC cells to elucidate the underlying mechanisms accounting for deactivation of NF-κB after treatment with AG. As shown in Figure 5A, AG strongly attenuated IKK-β phosphorylation in A549 and H1299 cells. We therefore speculated that AG could inactivate NF-κB by inhibiting IKK-β phosphorylation.

**Figure 6:**
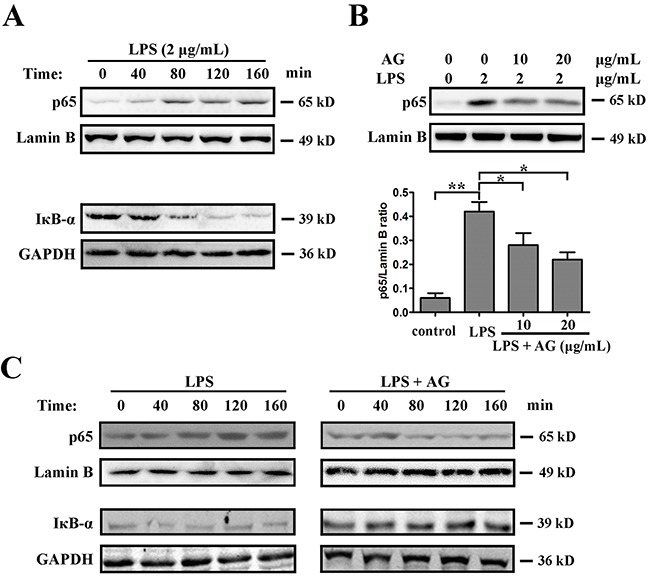
AG inhibits LPS-induced NF-κB activation **A**. A549 cells were cultured with 2 μg/mL LPS for 8 h. Nuclear proteins were extracted and subjected to Western blotting for p65 detection. Lamin B was used as loading control. Additionally, the whole cell extracts with the same treatment were prepared and analyzed for IκBα expression. GAPDH was used as loading control. **B**. A549 cells were cultured with 2 μg/mL LPS in the absence and presence of 10-20 μg/mL AG for 8 h. Nuclear proteins were extracted and subjected to Western blotting for p65 detection. Lamin B was used as loading control. Band intensity was quantified by Image J software. Data are representative of three independent experiments. *p < 0.05, **p < 0.01. **C**. A549 cells were cultured with 2 μg/mL LPS in the absence and presence of 10 μg/mL AG for the indicated time. Nuclear proteins were extracted and subjected to Western blotting for p65 detection. Lamin B was used as loading control. Additionally, the whole cell extracts with the same treatment were prepared and analyzed for IκBα expression. GAPDH was used as loading control.

The NF-κB pathway provides a pivotal link between inflammation and cancer. Exposure to proinflammatory stimuli in the tumor microenvironment may result in the activation of NF-κB in cancer. LPS is a powerful activator of NF-κB that can induce the inflammatory response in tumor cells. Therefore, the effects of AG on NF-κB activation induced by LPS were explored. The results showed that, compared with the control group, LPS (2 μg/mL) significantly reduced IκBα protein levels and enhanced the translocation of NF-κB from cytoplasm to nuclear in a time-dependent manner (Figure 6A). However, pre-treatment with AG (10 or 20 μg/mL) suppressed nuclear translocation of NF-κB induced by LPS in both a dose- and time-dependent manner in A549 cells (Figure 6B, 6C).

### AG sensitizes NSCLC cells to TNFα-triggered apoptosis

**Figure 7:**
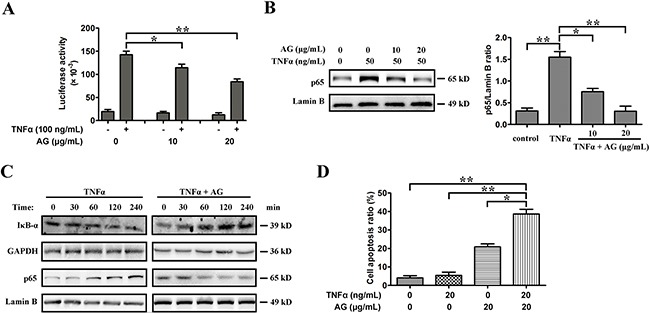
AG sensitizes A549 cells to TNFα-induced apoptosis by inhibiting NF-κB activity **A**. For NF-кB luciferase assay, cells were transiently transfected with the NF-кB luciferase reporter construct or empty vector. Transfected cells were treated with or without TNFα (100 ng/mL) in the presence or absence of different amounts of AG as indicated. NF-κB activities were determined by luciferase assays after 8 h of treatment. Data are representative of three independent experiments and are represented as mean ± SD. *p < 0.05, **p < 0.01. **B**. A549 cells were cultured with 50 ng/mL TNFα in the absence and presence of 10-20 μg/mL AG for 18 h. Nuclear proteins were extracted and subjected to Western blotting for p65 detection. Lamin B was used as loading control. Band intensity was quantified by Image J software. The results shown are representative of three different experiments. Data are represented as mean ± SD. *p < 0.05, **p < 0.01. **C**. A549 cells were cultured with 50 ng/mL TNFα in the absence and presence of 10 μg/mL AG for the indicated time. Nuclear proteins were extracted and subjected to Western blotting for p65 detection. Lamin B was used as loading control. Additionally, the whole cell extracts with the same treatment were prepared and analyzed for IκBα expression. **D**. A549 cells were treated with AG (20 μg/mL), TNFα (20 ng/mL), or their combination for 24 h before determination of cell death by flow cytometry analysis. Data are representative of three independent experiments and are represented as mean ± SD. *p < 0.05, **p < 0.01.

Although TNFα has cytotoxic effects on many tumors, it can also induce a marked inflammatory response *via* activating NF-κB. A number of tumor cells are resistant to TNFα, which is mostly due to NF-κB activation. To investigate whether AG treatment has an effect on TNFα-induced NF-κB activation, an NF-κB-dependent gene reporter assay was performed. A549 cells were transfected with an NF-κB-luciferase reporter construct transiently and then treated with TNFα. Treatment of cells with TNFα enhanced luciferase activity; however, AG attenuated the NF-κB activity induced by TNFα dose-dependently (Figure 7A). Further investigations into the molecular mechanism of action of AG showed that it inhibited the degradation of IκBα induced by TNFα and thereby blocked NF-κB (Figure 7B, 7C). Moreover, A549 cells were resistant to TNFα-triggered cell death at the concentration of 20 ng/mL. However, as shown in Figure 7D, AG and TNFα combinative treatment induced a synergistic increase in TNFα-triggered cell death (Figure 7D). These experiments suggested that AG could sensitize TNFα-induced apoptosis by suppressing NF-κB activity.

### Effects of AG on Fas/FasL-triggered apoptosis

**Figure 8:**
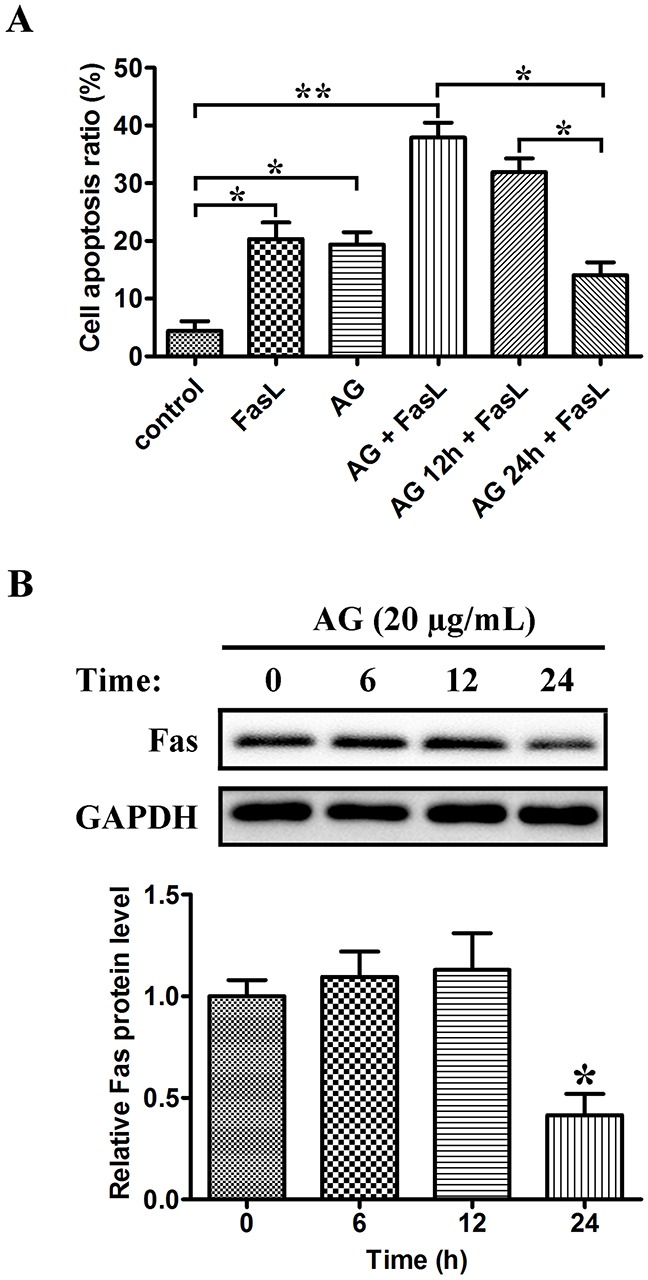
Dual effects of AG on Fas/FasL-induced apoptosis in A549 cells **A**. A549 cells were pre-treated with AG (20 μg/mL) for 0 h, 12 h, and 24 h, respectively, followed by the treatment with FasL (100 ng/mL) for 18 h. The cells were then analyzed for apoptosis by flow cytometry after Annexin V/PI staining. Data are represented as mean ± SD. *p < 0.05, **p < 0.01. **B**. A549 cells were treated with AG (20 μg/mL) for the indicated intervals (0 h, 6 h, 12 h, and 24 h). Western blotting was performed to examine the levels of Fas. Data are represented as the mean ± SD. *p < 0.05 compared with untreated cells.

Fas, also known as CD95, is a member of the TNF receptor family. FasL (also known as CD95L) is the corresponding ligand of Fas; binding of Fas by FasL results in apoptosis of Fas-expressing cells. A549 NSCLC cells have been reported to be sensitive to Fas/FasL-triggered apoptosis [20]. Therefore, we studied whether the sensitivity of A549 cells to FasL-induced apoptosis could be changed by AG pre-treatment at various intervals. As shown in Figure 8A, FasL and AG monotherapy induced apoptosis in 20.3% and 19.3% of cells respectively, whereas the combination of FasL and AG could induce apoptosis in 37.9% of cells. Similarly, pre-treatment with AG for 12 h followed by FasL treatment (18 h) produced 31.9% of apoptotic cells. Nevertheless, when cells were exposed to AG for 24 h, apoptosis induced by FasL was reduced to 14.1%. Western blotting was used to examine the expression of Fas in A549 cells following pre-treatment with AG. Figure 8B showed that A549 cells constitutively expressed Fas. The basal expression of Fas was significantly reduced after AG treatment for 24 h. These data suggested that AG changed the susceptibility of A549 cells to Fas/FasL-triggered apoptosis by altering the expression of Fas.

### AG retards the development of lung cancer xenografts in nude mice

**Figure 9:**
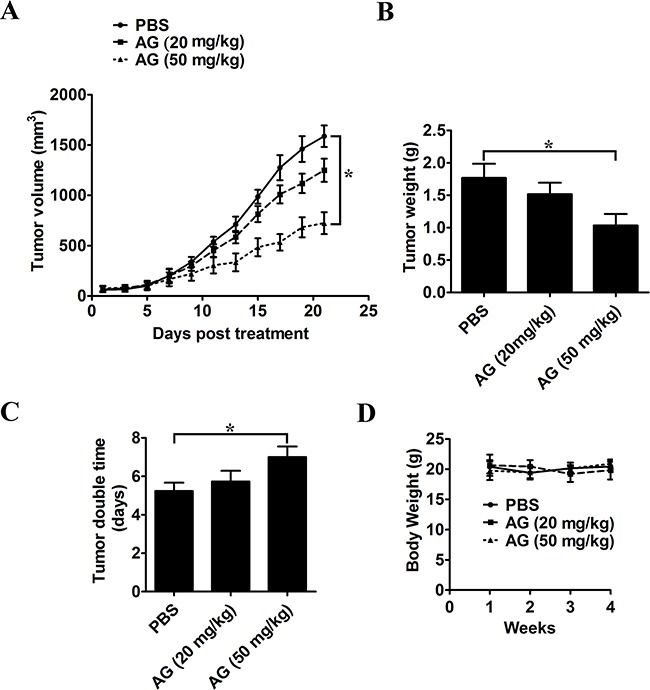
AG treatment inhibits *in vivo* tumor xenograft growth in a subcutaneous tumor model A549 cells were injected subcutaneously into the dorsal flanks of athymic nude mice. When tumors reached a size of approximately 50 mm^3^, mice were orally treated with AG at the dosage of 20 or 50 mg/kg every two day for a total of 21 days. **A**. The tumor growth inhibitory effects of different treatments were compared. **B**. At the end of the study, the excised tumors from each group were weighed. **C**. Tumor double time of each group. **D**. The weight of nude mice from each group did not change significantly during the experiment. All data are shown as mean ± SD. *p < 0.05.

A xenograft tumor model by transplanting A549 cells to nude mice was used to analyze the antitumor effects of AG. Before the tumor was palpated, mice were divided into three groups randomly on day 7 post-implantation. In each group, there were at least 8 tumor-bearing mice. Mice were treated orally with 20 or 50 mg/kg AG every two days for a total of 21 days. The growth of xenograft tumors was markedly suppressed in mice receiving treatment with 50 mg/kg AG (on days 13-21 vs. control; *p* < 0.05; Figure 9A). Tumors were removed and weighed at the end of the study. As compared with the control group, treatment with 50 mg/kg AG significantly reduced tumor weight (Figure 9B). The tumor doubling time was also extended from 5.23 days in mice treated with PBS, to 7.05 days in mice treated with 50 mg/kg AG (*p* < 0.05; Figure 9C). There were no apparent side effects, as expected. This was shown by a normal splenic structure (data not shown) and a continuous increase in body weight (Figure 9D).

**Figure 10:**
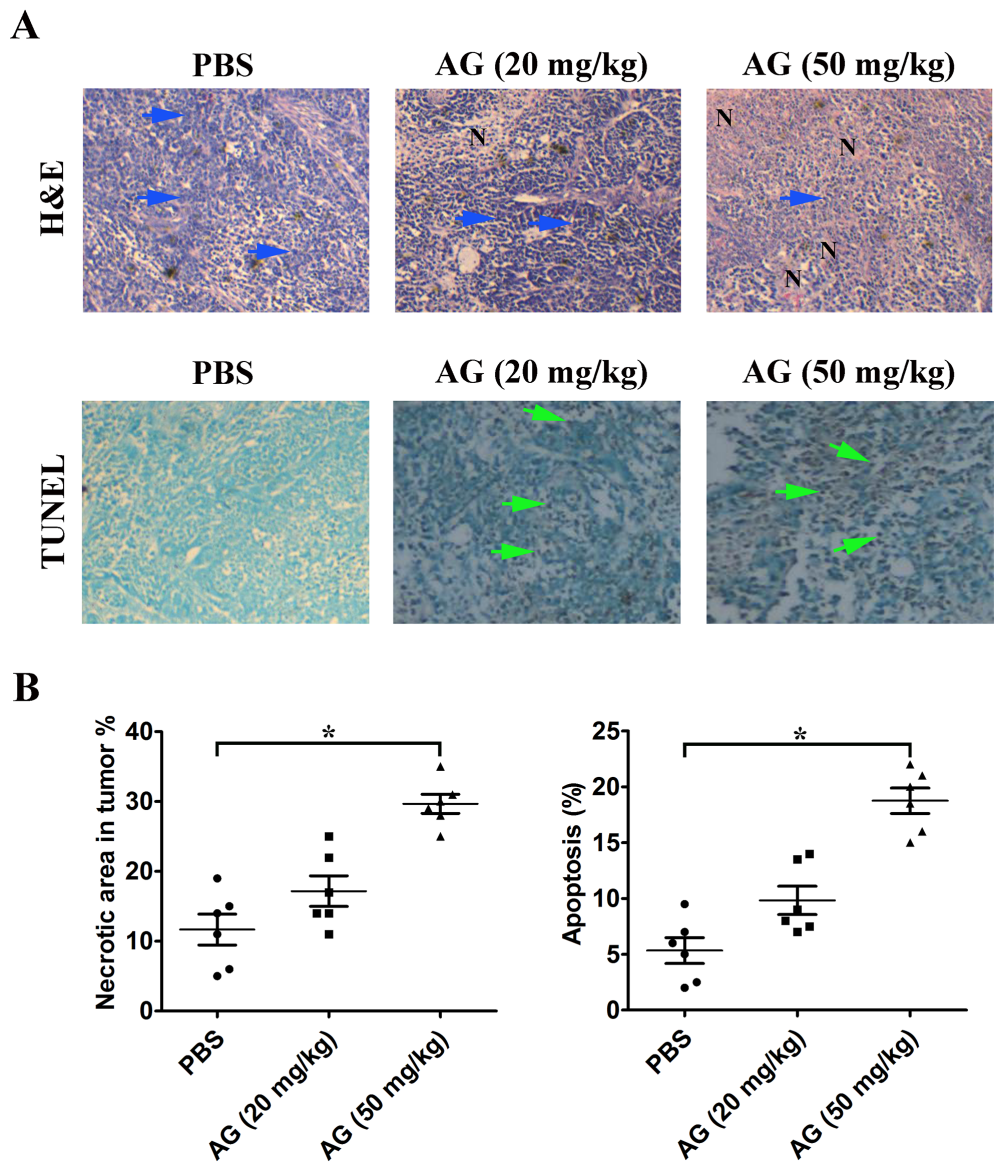
AG treatment induces necrosis of tumor and promotes tumor cell apoptosis **A**. Determination of tumor necrosis and apoptosis after treatment with AG. Tumor necrosis areas are shown by H&E staining and observed under light microscope (×100). The viable tumor cells are indicated by a *blue arrow*. TUNEL assay was used to detect apoptotic cells (original magnification, ×200). Positive cells for TUNEL staining are indicated by a *green arrow*. **B**. Quota of tumor necrosis and apoptosis. Tumor necrosis was determined by software Image J. Two sections/mouse and three mice were prepared (mean ± SD, *p < 0.05). The ratio of apoptotic cells to total cells: TUNEL positive cells were counted from three fields of the highest density of positive-stained cells in each section to determine the percentage of apoptotic cells (mean ± SD, *p < 0.05).

H&E staining indicated that tumor tissues from mice treated with AG had more severe necrosis than those that received the control treatment. The untreated control tumors showed tissue necrosis interspersed with viable cancer cells. The AG therapy, however, resulted in large areas of continuous necrosis within the tumor tissues (Figure 10A, 10B). The results of TUNEL assays further suggested that treatment with AG also induced programmed cell death *in vivo* (Figure 10A, 10B).

## DISCUSSION

As one of the most common causes of death worldwide, cancer takes almost 7 million lives each year. Despite advances in the development of new therapeutic modalities for cancer, chemotherapy is still a fundamental tool for cancer treatment primarily through induction of apoptosis in tumor cells. Various treatments for cancer are available; however, multi-drug resistance causes a rather low success rate in current chemotherapies, which emphasizes the importance of exploring new effective and safe antitumor compounds or drugs. Natural compounds isolated from medicinal plants are promising resources for the discovery of novel chemotherapeutic drugs [21–23]. Flavonoids are phenolic substances with potential antitumor function [24, 25]. One of these compounds, AG, a glycosidic form of kaempferol isolated from *Rosa agrestis*, has been described to exhibit anti-autophagy, anti-inflammatory, antioxidant, anti-apoptosis, and anti-atopic effects [11, 13, 26–28]. Cytotoxic activity of flavonoids was reported to be related to the origin of cancer cells [29]. AG was previously reported to exert highly cytotoxic effects on HepG2 cells [30]. Kaempferol, which is a phytoestrogen belonging to the flavonoids mostly found in fruits and plants, has activity against many types of cancer *via* regulating a variety of cancer cell features, such as cell cycle [31], inflammation [32, 33], and apoptosis [34, 35]. Kaempferol has also been found to exhibit anti-oxidant and cellular membrane protective effects [36]. However, the efflux of kaempferol restricts its potential use as an anticancer agent [37, 38]. Therefore, in the current study, we studied whether the glycoside of kaempferol, AG, has a remarkable bio-activity in lung cancer using a pair of NSCLC cell lines.

MTT assays revealed that AG inhibited the proliferation of tumor cells significantly. Apoptosis is a form of programmed cell death that involves several signaling pathways. To investigate the anticancer mechanism of action of AG, the activation of caspases-8, -9, and -3, as well as NF-κB/p65 activity were measured. The data indicated that AG induced apoptosis in tumor cells *via* the activation of caspase-9/caspase-3 and the inhibition of NF-κB/p65. The activation of caspases subsequently led to the cleavage of PARP, nuclear condensation, and finally, the induction of apoptotic cell death. The Bcl-2 family proteins play anti-apoptotic (Bcl-xl, Bcl-2) or pro-apoptotic (Bid, Bak, Bax) roles in the mitochondrial apoptosis pathway by regulation on the mitochondrial membrane permeability. It is well-known that upregulation of Bax:Bcl-2 ratio will lead to the discharge of some pro-apoptotic proteins from the mitochondria [39]. Conversely, the elevated expression of some anti-apoptotic proteins, such as Bcl-xl and Bcl-2, prevents apoptosis of cancer cells [40]. In this study, AG treatment upregulated Bax and Bad with concomitant downregulation of Bcl-2 and Bcl-xl; this caused upregulation of the Bax:Bcl-2 ratio and triggered the activation of mitochondria-dependent caspase cascade to induce apoptotic cell death in A549 and H1299 cells. In support of the pro-apoptotic effects of AG, Burmistrova *et al*. [41] have reported that, in human leukemia cells, AG heptaacetate induced cell death including apparent caspases activation and a marked upregulation of the Bax:Bcl-2 ratio.

The MAPK (mitogen-activated protein kinase) family consists of a series of serine/threonine kinases playing critical roles in control of growth and differentiation, as well as in apoptotic signaling, which include members such as ERK 1/2, p38, and JNK. The exact signaling pathways among three types of MAPKs are still unclear; however, ERK 1/2 is thought to play an important role in survival [42], whereas in a number of cell types, JNK has been reported to be associated with proapoptotic actions [42]. MAPK signaling cascades have also been demonstrated to be involved in NF-κB activation [43]. In our study, AG treatment resulted in JNK activation; however, it inhibited PI3K/Akt activation, as well as p38 and ERK1/2 phosphorylation. Therefore, combinative treatment with AG and Akt and/or MEK1/2 inhibitors might be a potential therapeutic strategy in clinical therapy. As expected, our data indicated that NSCLC cell apoptosis was enhanced when the PI3K/Akt or ERK1/2 pathway was blocked. This suggests that AG plays a central apoptotic role in tumor cells, probably by inhibiting MAPK signaling pathways. Previous studies demonstrated that AG heptaacetate activated ERK1/2 and p38 in human leukemia cells [41]. In our research, we found that AG decreased ERK1/2 and p38 phosphorylation in NSCLC cells. This might be explained by the fact that the current study and the report by Burmistrova *et al*. [41] used different cell types, and that the compounds used differed slightly.

A main cause of cancer is inflammation. The activation of NF-κB, which is a principle indication of inflammatory response and is often observed in malignant tumors including breast, ovarian, and lung cancer [44, 45], could be a missing link between inflammation and cancer [46]. In most resting cells, NF-κB exists in the cytosol as an inactive form associated with an inhibitory protein termed IκB. Once cells are exposed to inflammatory stimuli, e.g., LPS and TNFα, IκB is phosphorylated by the upstream kinase IKK and subsequently degraded, leading to the release and nuclear translocation of NF-κB [47]. The activated NF-κB heterodimers then binds to consensus sequences activating expression of target genes [48]. LPS, a component of the Gram-negative bacterial cell wall, can induce strong proinflammatory response in cells and animals [49]. The binding of LPS to its receptor TLR4 results in the activation of IKK, the subsequent degradation of IκBα and NF-κB activation. The data presented herein indicated that NF-κB was activated in A549 cells treated by LPS. Moreover, AG inhibited LPS-induced activation of NF-κB by inhibiting IκBα degradation dose-dependently. These results strongly imply that AG exhibits potent anti-inflammatory function and that it might represent a novel strategy for cancer therapy.

Resistance to apoptosis rescues tumor cells from death and is critical for cancer cell survival. Thereafter, targeting the apoptotic pathway is a main strategy for antitumor therapy. The death receptors pathway can be induced by ligation of a subset of plasma membrane tumor necrosis factor receptor (TNFR) family members, such as the receptors for FasL and TNFα [50]. Strategies that target the death receptors signaling have been proposed and investigated for the therapy of many cancers [51]. TNFα has previously been utilized in antitumor therapies because of its cytotoxic effects on tumor cells [52]. However, the clinical advantages of TNFα are limited because it induces a profound inflammatory response by activating NF-κB [53]. Previous studies have demonstrated that, in NSCLC cell lines including A549 cells, NF-κB was activated by TNFα, which promoted cell survival, angiogenesis, and invasion [54–56]. NF-κB activity largely controls the decision between life and death with TNFα treatment. Therefore, suppressing TNFα-induced NF-κB signaling can potentiate TNFα-induced apoptosis [44]. Our study demonstrated that AG attenuated NF-κB activity and induced a synergistic increase in apoptosis in A549 cells treated with TNFα. In accord with past reports, the inhibition of NF-κB activation led to increased TNFα-induced apoptosis [57]. Moreover, AG reduced TNFα-stimulated IκBα degradation, which suppressed the nuclear translocation of NF-κB. These data suggest that AG can sensitize TNFα-induced apoptosis and reduce the pro-inflammatory side effects by suppressing the NF-κB activity. Therefore, AG may be used as a TNFα adjuvant for cancer therapy.

FasL is a transmembrane protein that belongs to the TNF family. The FasL-Fas association plays a critical role in the regulation of various biological responses [58]. Interestingly, the current study indicated that A549 cells exhibited opposing responses to FasL-triggered apoptosis following AG treatments at various intervals. As compared with cells receiving FasL or AG monotherapy, cell apoptosis increased about two-fold after combinative treatment with AG and FasL, or pre-treatment with AG for 12 h. Nevertheless, FasL-induced apoptosis in A549 cells decreased markedly after pre-treatment with AG for 24 h. Consistent with this, the reduction in FasL-triggered apoptosis was concomitant with the decrease of Fas expression in A549 cells following AG pre-treatment for 24 h. Together, these results presented here suggest that AG could alter the sensitivity of A549 cells to Fas/FasL-induced apoptosis time-dependently.

Similar results were observed *in vivo*. Using a tumor xenograft model, we found that AG-triggered cancer cell apoptosis also occurred *in vivo*, and that 50 mg/kg AG efficiently inhibited tumor growth. According to the H&E and TUNEL staining results, the most effective suppression of tumor growth was attributable to apoptosis induced by 50 mg/kg AG. The novel antitumor effect of AG in NSCLC cells *via* the inhibition of MAPKs and NF-κB activity suggest that AG could be used as a promising drug for cancer therapy.

**Figure 11:**
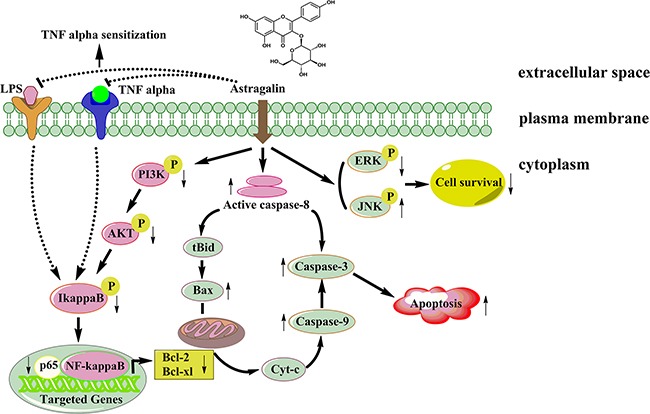
A proposed signaling pathway by which AG induces cell death in NSCLC cells AG treatment inhibits Akt phosphorylation *via* the PI3K; this inhibits IκBα phosphorylation and degradation, suppresses the translocation of p65, and, in turn, decreases the Bcl-2 and Bcl-xl expression, leading to apoptosis of NSCLC cells. AG treatment also inhibits LPS- or TNFα-induced NF-κB activation, sensitizing NSCLC cells to TNFα-induced apoptosis. In addition, AG treatment activates JNK, but inhibits ERK signaling, which finally leads to decreased cell survival.

In conclusion, our study showed, for the first time, the apoptotic effect of AG against NSCLC cells. Four novel findings may account for this role of AG treatment: (1) activation of caspases; (2) alteration of the Bax:Bcl-2 ratio; (3) reduction of LPS- or TNFα-induced nuclear translocation of NF-кB; and (4) inhibition of MAPKs and PI3K/Akt pathway (Figure 11). AG treatment to control the growth of cancer cells might be a potential therapeutic strategy. It may circumvent adverse side effects and drug resistance that frequently occurred in current cancer therapy. In the coming years, the promising anticancer mechanism of AG should be further studied to establish a powerful therapeutic strategy for successful therapy of human cancer. However, further *in vivo* pharmacological and clinical investigations are required.

## MATERIALS AND METHODS

### Cells, cell culture, and reagents

NSCLC cell lines A549 and H1299, and human bronchial epithelial cells (BEAS-2B cells) were purchased from the American Type Culture Collection (ATCC, Philadelphia, PA, USA). NSCLC cells were grown in RPMI 1640 (Invitrogen, Carlsbad, CA, USA) supplemented with 10% (v/v) fetal bovine serum (FBS; Invitrogen, Carlsbad, CA, USA) and 1% penicillin-streptomycin (Invitrogen, Carlsbad, CA, USA). BEAS-2B cells were cultured following standard guidelines. Thawed cells initially grew in a pre-coated flask containing fibronectin (0.01 g/mL), bovine collagen type I (0.03 mg/mL), and bovine serum albumin (0.01 mg/mL). Following overnight growth in this pre-coated flask, BEAS-2B cells were sub-cultured in DMEM (Invitrogen, Carlsbad, CA, USA) supplemented with 10% FBS, 100 U/mL penicillin and 100 U/mL streptomycin (Invitrogen, Carlsbad, CA, USA). All cells were cultured in a humidified CO_2_ incubator at 37°C. MEK inhibitor AZD6244 (Calbiochem, Merck Biosciences, Darmstadt, Germany), PI3K/Akt inhibitor LY294002 (Sigma, St. Louis, MO) were dissolved in dimethyl sulfoxide (DMSO) and freshly prepared each time before use. AG (purity > 95 %) was purchased from the National Institutes for Food and Drug Control (Beijing, China), and was dissolved in DMSO for live culture with cells; a final culture concentration of DMSO was ≤ 0.5%. LPS (Escherichia coli 055:B5) was purchased from Sigma Chemical Co. (St. Louis, MO, USA). PI was purchased from Molecular Probes (Eugene, OR, USA).

### Flow cytometry

Cells were treated 0-20 μg/mL AG in the absence or presence of either TNFα (20 ng/mL), or AZD6244 (2 μM), or LY294002 (5 μM). 18 h after treatment, the ratio of cell apoptosis was determined by flow cytometry analysis as previously described [59].

### Cell proliferation assay

The effects of AG on cell proliferation were assessed by the MTT assay. Cells in the exponential growth phase were seeded into a 96-well plate at a density of 5000 cells per well. After 24 h, 0-40 μg/mL AG was added to the medium. The cells were incubated at 37°C for 24 h, then the cell viability was determined by the colorimetric MTT [3-(4, 5-dimethylthiazol-2-yl)-2, 5-diphenyl-2H-tetrazolium bromide] assay at wave length 570 nm by TECAN Safire Fluorescence Absorbance and Luminescence Reader (Vienna, VA, USA). The cell viability was calculated according to the formula: Cell viability (%) = average A_570 nm_ of treated group/average A_570 nm_ of control group × 100%. Each experiment was performed in quadruplicate and repeated at least three times.

### Colony-forming assay

Colony-forming assay was performed as previously described [59]. Briefly, about 300 cells in log phase were plated into 60 mm tissue culture Petri-dish (Greiner) in triplicate with 3 mL of culture medium and grown at 37°C with 5% CO_2_. After 48 h culture for cell adherence to the plate, rinsed with fresh medium, AG was added at different concentrations (0, 20, 40 μg/mL). 48 h later, the cells were washed twice with PBS and then incubated in drug-free medium. The medium was changed every 5 days. After culturing for additional 10-14 days, the medium was discarded and each dish was washed twice with PBS carefully. The cells were fixed with methanol for 15 min and stained with a 1:10 dilution of Giemsa regent (Merck, Germany) for 10 min. Any grouping of cells containing 30 or more cells was counted as a colony using an inverted microscope (Zeiss, 40-fold magnification). Colony numbers were determined from triplicate plates. Colony growth was related to the control value without any treatment.

### Caspase activity assay

Caspase-3 and caspase-9 activities were measured using colorimetric activity assay kits (Chemicon International, CA, USA). The assay is based on the cleavage of the chromogenic substrates, DEVD-pNA and LEHD-pNA, by caspase-3 and caspase-9, respectively. Cells were lysed in chilled lysis buffer on ice for 10 min and centrifuged for 5 min at 10,000 × *g*. Caspase substrate solution containing the specific peptide substrate was then added to the supernatant and incubated for 2 h at 37°C before measurement by ELISA reader at 405 nm

### Western blot analysis

Whole cell lysates were prepared in 1 mM Tris-HCl (pH 6.8) lysis buffer containing 10% SDS, 1% glycerophosphate, 0.1 mM Na_3_VO_4_, 0.5 mM NaF and protease inhibitor cocktail. Supernatants were collected and protein concentration was determined by the Bio-Rad protein assay method (Bio-Rad, Hercules, CA). In addition, nuclear extracts were prepared as described by Schreiber *et al* [60]. Western blotting used standard protocols. Proteins were separated by SDS-PAGE and transferred onto nitrocellulose membranes that were blocked with 5% non-fat milk in TBS containing 0.1% Tween-20, and incubated with primary antibodies: p-JNK, JNK, Akt, p-Akt (Ser 473), PI3K, p-PI3K, Fas, cleaved caspase-9, cleaved caspase-8, cleaved caspase-3 (Cell Signaling Technology, Beverly, MA, USA), NF-κB p65 (Invitrogen, Carlsbad, CA), p-IKK-β, IкBα, p-IкBα (Sigma, St. Louis, MO), GAPDH, Lamin B, ERK, p-ERK, p-p38, p38, IKK-β, poly (ADP-ribose) polymerase (PARP), Bad, Bax, Bcl-xl, Bcl-2 (Santa Cruz Biotechnology, Santa Cruz, CA). Secondary antibodies were coupled to horseradish peroxidase, and were goat anti-rabbit or goat anti-mouse. Bound antibodies were then visualized with ECL plus Western blotting detection reagents (GE Healthcare). Signal intensity was quantified by densitometry using the software Image J (NIH, Bethesda, MD). All experiments were done in triplicate and performed at least three times independently.

### DAPI staining

DAPI staining was applied for morphological assessment of nuclei. Treated A549 cells in 6-well plate were rinsed twice with cold PBS and stained with DAPI solution (1 μg/mL) for 10 min at 37°C in dark room. Stained cells were washed twice with cold PBS. Finally, an inverted fluorescence microscopy (CKX41, OLYMPUS, Japan) was used to photograph the cells in the plate. At least 200 cells were counted and classified according to the condensed nuclei.

### TUNEL assays

Exponentially growing cells were treated with 0-20 μg/mL AG for 24 h. The TdT-mediated dUTP nick end labeling (TUNEL) assay was performed as previously described [59].

### Transient transfection and luciferase activity assay

For transient transfections, A549 cells were seeded at 2 × 10^6^ in a 6-well plate one day before transient transfection. The expression vector containing NF-кB luciferase reporter construct (pNF-κB-LUC, plasmid containing NF-кB binding site) were transfected with serum- and antibiotics-free RPMI 1640 medium containing 8 μL of Lipofectamine 2000 reagent (Invitrogen, Carlsbad, CA, USA). After 5 h of incubation, medium was replaced with RPMI 1640 medium containing 10 % FBS and antibiotics. Cells were allowed to recover at 37°C for 20 h and subsequently were stimulated as indicated. For luciferase activity assay, cell lysates were prepared and measured for luciferase activity using Luciferase Assay System (Promega, Madison, WI, USA), according to the manufacturer's instructions.

### Animals

Athymic nude mice (6-8 weeks of age) were obtained from Shanghai Laboratory Animal Center (Shanghai, China) and housed under germfree conditions. Animal care and use were performed strictly in accordance with the ethical guidelines by the Nanjing University Animal Care and Use Committee, and the study protocol was approved by the local institution review board. The animals were randomly allocated into experimental groups. The blinding process was employed in animal experiments.

### *In vivo* animal tumor model experiment

A549 cells (5×10^5^ cells in 30 μL) were injected subcutaneously into the dorsal flanks of mice. Tumor volume was monitored by measuring the two maximum perpendicular tumor diameters with callipers every other day. All tumor-bearing mice were divided randomly into three groups, and treatment was initiated on the 7^th^ day when the volume of tumor reached a size of approximately 50 mm^3^. The mice were treated with AG (20 or 50 mg/kg, administered by gavage) every two days for a total of three weeks. Control mice received treatment of PBS. Antitumor activity of treatments was evaluated by tumor growth inhibition. The formula, tumor volume = length × width^2^ × 0.52, was used to mimic the tumor volume. At the end of study, the tumors were collected and weighed.

### H&E and TUNEL assays

In a parallel animal assay (totally three groups, and three mice per group), the tumor establishment and drug treatment are the same as described above. On the 21^th^ day, mice were euthanized. Tumors were collected, fixed with 4% formaldehyde, embedded in paraffin. Tissue sections (5 μm in thickness) were prepared according to standard protocols for hematoxylin/eosin (H&E) staining. Apoptotic cells in tumor sections (two sections per mouse, three mice in total) were visualized by the TUNEL technique according to the manufacturer's instruction (Merck).

### Statistical analysis

Statistical analysis was carried out using the SPSS software (version 11.0; SPSS, Chicago, IL). Data were expressed as the mean ± standard deviations (SD). For paired data, statistical analyses were performed using two-tailed Student's t-tests. For multiple comparisons, statistical analyses were performed using one-way analysis of variance (ANOVA) with a Tukey post-test. For all analyses, *P* < 0.05 was considered statistically significant.

## SUPPLEMENTARY FIGURES



## Abbreviations

AG: astragalin
TNFα: tumor necrosis factor alpha
AIF: apoptosis-inducing factor
PARP: poly (ADP-ribose) polymerase
NSCLC: non-small cell lung cancer
LPS: lipopolysaccharide
DMSO: dimethyl sulfoxide
PI: propidium iodide
IκB: the inhibitor of κB
IKK: IκB kinase
MAPK: mitogen-activated protein kinase
ERK: extracellular signal-regulated kinase
JNK: c-Jun N-terminal kinase
TRAIL: TNF-related apoptosis-inducing ligand
DR: death receptor
FasL: Fas ligand
