# Supplementary material for: Astragalin-induced cell death is caspase-dependent and enhances the susceptibility of lung cancer cells to tumor necrosis factor by inhibiting the NF-κB pathway
Source: Oncotarget. 2017 Feb 10;8(16):26941–58. doi: 10.18632/oncotarget.15264 (PMC5432309; doi:10.18632/oncotarget.15264)
Supplement: Supplementary file 1 [file oncotarget-08-26941-s001.pdf]

Astragalin-induced cell death is caspase-dependent and enhances the susceptibility of lung cancer cells to tumor necrosis factor by inhibiting the NF-κB pathway

SUPPLEMENTARY FIGURES

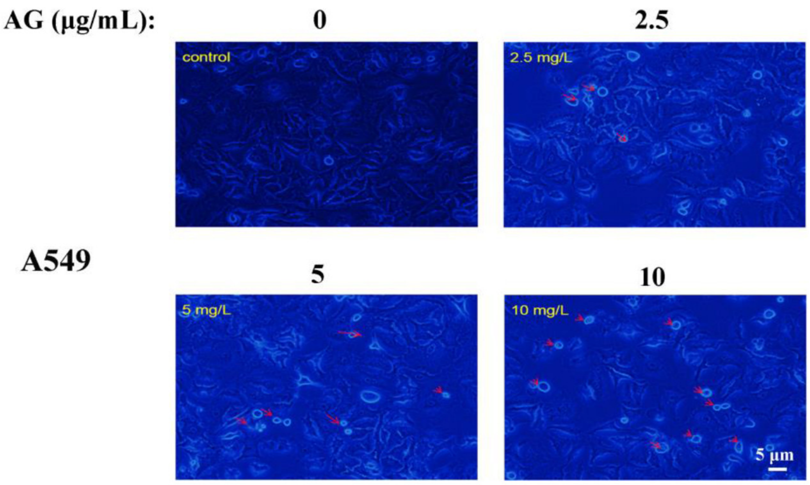

Supplementary Figure 1: A549 cells were exposed to AG at different concentrations (0, 2.5, 5, 10 μg/mL) for 24 h. DAPI staining in treated groups in A549 cells were shown: apoptotic cells show condensed nuclei with karyorrhexis which are stained bright blue (Arrows).

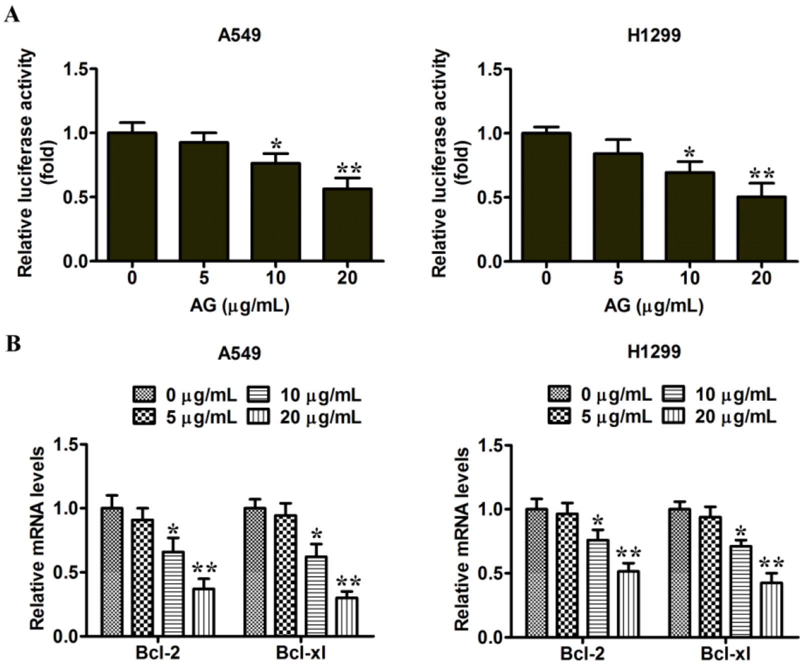

Supplementary Figure 2: Effects of AG on NF-κB activity and *Bcl-2* and *Bcl-xl* mRNA levels. A. For NF-κB luciferase assay, cells were transiently transfected with the NF-κB luciferase reporter construct or empty vector. Transfected cells were treated with different amounts of AG as indicated. NF-κB activities were determined by luciferase assays after 8 h of treatment. Data are representative of three independent experiments in triplicate and are represented as mean ± SD. *$p < 0.05$, **$p < 0.01$. **B.** A549 cells were treated with different amounts of AG as indicated for 24 h. Q-PCR analysis was performed to detect the level of the mRNA transcripts of *Bcl-2* and *Bcl-xl*. The results shown are representative of three independent experiments. The histogram shows the mean ± SD. *$p < 0.05$, **$p < 0.01$.
